# Antibody Therapies for Acute Myeloid Leukemia: Unconjugated, Toxin-Conjugated, Radio-Conjugated and Multivalent Formats

**DOI:** 10.3390/jcm8081261

**Published:** 2019-08-20

**Authors:** Brent A. Williams, Arjun Law, Judit Hunyadkurti, Stephanie Desilets, Jeffrey V. Leyton, Armand Keating

**Affiliations:** 1 Cell Therapy Program, Princess Margaret Cancer Centre, Toronto, ON M5G 2C1, Canada; 2 Hans Messner Allogeneic Blood and Marrow Transplant Program, Princess Margaret Cancer Centre, Toronto, ON M5G 2C1, Canada; 3 Département de medécine nucléaire et radiobiology, Faculté de medécine et des sciences de la santé, Centre hospitalier universitaire de Sherbrooke (CHUS), Université de Sherbrooke, Sherbrooke, QC J1H 5N4, Canada; 4 Service de hemato-oncologie, CHUS, Sherbrooke, QC J1H 5N4, Canada; 5 Sherbrooke Molecular Imaging Centre, Centre de recherche du CHUS, Sherbrooke, QC J1H 5N4, Canada; 6 Institute de pharmacologie de Sherbrooke, Université de Sherbrooke, Sherbrooke, QC J1H 5N4, Canada

**Keywords:** acute myeloid leukemia, AML, antibody, bi-specific antibody, therapy

## Abstract

In recent decades, therapy for acute myeloid leukemia (AML) has remained relatively unchanged, with chemotherapy regimens primarily consisting of an induction regimen based on a daunorubicin and cytarabine backbone, followed by consolidation chemotherapy. Patients who are relapsed or refractory can be treated with allogeneic hematopoietic stem-cell transplantation with modest benefits to event-free and overall survival. Other modalities of immunotherapy include antibody therapies, which hold considerable promise and can be categorized into unconjugated classical antibodies, multivalent recombinant antibodies (bi-, tri- and quad-specific), toxin-conjugated antibodies and radio-conjugated antibodies. While unconjugated antibodies can facilitate Natural Killer (NK) cell antibody-dependent cell-mediated cytotoxicity (ADCC), bi- and tri-specific antibodies can engage either NK cells or T-cells to redirect cytotoxicity against AML targets in a highly efficient manner, similarly to classic ADCC. Finally, toxin-conjugated and radio-conjugated antibodies can increase the potency of antibody therapies. Several AML tumour-associated antigens are at the forefront of targeted therapy development, which include CD33, CD123, CD13, CLL-1 and CD38 and which may be present on both AML blasts and leukemic stem cells. This review focused on antibody therapies for AML, including pre-clinical studies of these agents and those that are either entering or have been tested in early phase clinical trials. Antibodies for checkpoint inhibition and microenvironment targeting in AML were excluded from this review.

## 1. Introduction

The discovery of a means to generate murine monoclonal antibodies by George Köhler and César Milstein garnered the 1984 Nobel Prize in Medicine and paved the way for a new class of therapeutics [1]. Monoclonal antibodies (mAbs) have transformed therapy for numerous diseases, including cancer. Rituximab (anti-CD20 chimeric antibody) was the first monoclonal antibody approved for use in cancer and tested experimentally in a clinical trial for lymphoma in 1998 [2]. The approach to the antibody therapy of cancer has developed rapidly, leading to several general therapeutic approaches: (1) unconjugated classical antibodies (e.g., rituximab/Rituxan), (2) toxin-conjugated antibodies (e.g., gemtuzumab ozogamicin (GO)/Mylotarg), (3) bi- and tri-specific recombinant antibodies (e.g., blinatumomab/Blincyto), and 4) radio-conjugated antibodies (e.g., ^131^I-BC8/Iomab-B). Acute myeloid leukemia (AML) represents a challenging malignancy to treat, particularly in the situation of relapsed and refractory AML (R/R AML), and antibody therapeutics have not, in general, become a standard of care for most patients. In this review, we will discuss basic aspects of AML biology, which inform the strategies that have been used in developing targeted antibody therapies to complement, enhance or replace existing standards of care. We highlighted four major approaches to antibody therapy, emphasizing mechanisms of cytotoxicity and data from both pre-clinical and clinical studies of specific agents. We will not cover checkpoint inhibitors that facilitate T-cell anti-tumour responses (e.g., ipilimumab/Yervoy; anti-CTLA-4), or similar approaches to facilitate macrophage anti-tumour responses (i.e., Hu5F9-G4; anti-CD47), or antibodies not directed at cancer cells specifically, such as those that target stroma (BMS-936564/MDX1338/ulocuplumab; anti-CXCR4).

### 1.1. Current Standard of Care for AML

The core of most AML chemotherapy regimens consists of continuously infused cytarabine with anthracyclines such as daunorubicin [3]. The most frequently used regimen, referred to as “3 + 7”, consists of continuous infusion of cytarabine at 100 mg/m^2^ for seven days and rapid intravenous injection of daunorubicin on the first three days of the treatment cycle [4]. Another subsequent trial showed improved complete response (CR) rates, with 45 mg/m^2^ of daunorubicin over lower doses, particularly in younger patients [5]. Dose intensification of cytarabine and daunorubicin at 45 or 90 mg/m^2^ for 3 days was assessed in a large prospective trial showing that adverse events were similar in both arms and a significantly higher complete remission rate was achieved with higher dosing of daunorubicin (67.6% vs. 57.2%). However, in this study, OS was only significantly improved in patients with favorable or intermediate risk cytogenetics [6]. Further studies also confirmed the improved efficacy of high dose daunorubicin (90 mg/m^2^), including subgroup analysis that showed superior outcomes in patients with mutations in *DNMT3A*, *NPM1*, and *MLL* [7,8,9]. The UK National Cancer Research Institute AML17 trial compared an intermediate 60 mg/m^2^ dose to 90 mg/m^2^ and found no differences in CR rate but a higher dose arm was associated with greater mortality at day 60 [10]. Current recommendations suggest that daunorubicin doses should be ≥60 mg/m^2^ in all cases [11].

Several attempts have been made to improve upon the success of induction chemotherapy by adding other agents, but none have shown significant improvements in outcomes without increasing toxicity. The topoisomerase II inhibitor, etoposide, has single agent activity against AML and has been incorporated into induction or consolidation protocols depending on the risk category, age, and cardiac status of the patient [12]. However, there are no data to suggest that adding etoposide or 6-thioguanine to 3 + 7 improves outcomes [13,14]. More intense combination regimens, such as FLAG-Ida (Fludarabine, Cytarabine, Idarubicin, and Filgrastim), have higher rates of CR but are associated with increased toxicity, resulting in no improvement in overall survival [15,16].

A risk-adapted approach may be beneficial in certain situations. AML with *FLT3*-ITD mutations may benefit from the addition of targeted therapy with tyrosine kinase inhibitors (TKIs) like midostaurin [17]. Similarly, the addition of GO has improved outcomes in patients with favourable risk of AML with core binding factor mutations [18]. More recently, a specialized liposomal formulation containing cytarabine and daunorubicin (CPX-351/Vyxeos) was evaluated in patients with R/R-AML, and later, in patients with de novo high-risk or secondary AML, with improvements in survival in both settings [19,20,21]. This drug is now approved for use in therapy-related AML and AML with myelodysplasia-related changes [22,23].

Furthermore, patients unable to tolerate standard chemotherapy have benefited from venetoclax, an oral B-cell leukemia/lymphoma-2 (BCL-2) inhibitor with a 19% overall response rate and an additional 19% demonstrating partial anti-leukemic activity [24]. A subsequent phase 1b study of venetoclax in patients ≥65 years of age with treatment-naive AML ineligible for standard chemotherapy who received oral venetoclax in combination with the hypomethylating agents decitabine or azacytidine resulted in a 67% CR or Cri, maintained with a median duration of 11.3 months [25].

Virtually all patients who achieve CR relapse without post-remission therapy [26]. Consolidation chemotherapy with high-dose cytarabine (HiDAC) was found to be effective in preventing relapse in up to 44% of patients who did not receive allogeneic hematopoietic stem-cell transplantation (HSCT) [27]. While there are significant variations in doses between centers, a dose of 3 g/m^2^ administered on days 1, 3, and 5 of each course was considered optimal [28]. Finally, it has been well established that allogeneic HSCT is the post-remission treatment of choice in eligible patients with intermediate or high-risk AML [29].

However, despite these therapies, five-year survival for AML patients was approximately 40% in adults [30] and 60% in children [31], with high-risk groups faring much worse (<10%) [32]. Survival outcomes remained particularly poor for patients over the age of 60 [33]. To improve outcomes, novel therapeutics are needed and antibody-based therapeutics have the potential to integrate with currently used standard-of-care regimens. However, selecting the right antibody-based treatment strategy in combination with complementary AML biological aspects, such as target antigens and leukemogenic stem cells, is key to providing patients with long-term leukemic-free survival.

### 1.2. AML Cell Surface Antigens

AML cells typically express antigens found on normal myeloid progenitor and differentiated cells, such as macrophages and monocytes, with aberrant expression of other lineage markers. AML expresses the pan-leukocyte marker CD45 and other myeloid markers such as CD11b, CD13 and CD33. A review of 106 adult AML cases was conducted to assess immunophenotypic variation based on the French-American-British (FAB) classification using a 22-antibody panel [34]. The most commonly expressed antigens were CD45 (97.2%), CD33 (95.3%), and CD13 (94.3%). Lymphoid-associated antigens were expressed in approximately half of cases, with the following descending order of frequency: CD20 (17%), CD7 (16%), CD19 (9.8%), CD2 (7.5%), CD3 (6.7%), CD5 (4.8%), and CD10 (2.9%). CD56, typically found on NK cells, can also be found on AML cells, but not on normal myeloid cells. CD56 expression in t(8;21) AML was associated with a higher rate of relapse [35]. These markers provide potential therapeutic targets to exploit.

### 1.3. Cancer Stem Cell Hypothesis and Optimal Antigen Targets in AML

Normal hematopoietic stem cells were first discovered by Till and McCulloch [36], which led them to explore the underlying process that determined cell fate decision to self-renew or differentiate based on stochastic or deterministic models. Ultimately, hematopoietic stem cells (HSCs) were identified as being enriched in the CD34 + CD38- fraction of bone marrow cells and cord blood [37] and capable of recapitulating an entire hematopoietic system in both animal and human models. The concept of a cancer stem cell in leukemia was proposed by Bruce et al. [38] and provided an alternative to the stochastic model to explain rare tumour-initiating cells. One additional feature of this theory was that cancer cells could differentiate along a hierarchy somewhat analogous to normal HSCs. Leukemia stem cells (LSCs) in AML were the first identified cancer stem-cell population, which were shown to be enriched in the CD34 + CD38- fraction of whole blasts, as measured by the ability to engraft in the bone marrow of immunodeficient mice [39,40]. Most subsequent studies of leukemic stem cells (LSCs) have focused on the CD34 + CD38- definition with or without addition of CD123 or CLL-1 [41,42,43], and these have informed the majority of therapeutic strategies to date that target LSCs. Other markers that are expressed on LSCs and have utility in further discriminating them from HSCs include CD7, CD32, CD45RA, CD96, CD99, CD157, CD244, IL-1 receptor accessory protein (IL-1RAP) [44], and T cell immunoglobulin mucin-3 (TIM-3), which are discussed briefly below. However, some of these antigens are expressed on normal tissues and might pose challenges to target therapeutically. CD7 and CD96 were both found on T and NK cells populations [45,46], CD32 on myeloid cells and B-cells [47], CD45RA on naïve T-cells [48], CD99 on all leukocytes [49], CD244 on NK and T cells [50], CD157 on all myeloid progenitors, neutrophils and macrophage [51], IL-1RAP on neutrophils, monocytes and lymphocytes [52] and TIM-3 on T, NK, macrophage and dendritic cells [53]. CD96 has been shown to be expressed on the majority of AML LSCs, with minimal expression on HSCs making it a potential target [54]. TIM-3 is expressed on LSCs in most types of AML, but is absent on HSCs and can be therapeutically targeted in AML xenograft models with TIM-3 specific antibodies [55]. CD157 has been shown to be present on 97% of primary AML blasts, with some expression on LSC sub-populations and a novel humanized antibody has been generated with in vitro activity in combination with NK cells [56]. CD244 has been implicated in maintaining and driving LSCs and represents an emerging target [57].

Finally, several lines of evidence support the clinical relevance of what is commonly referred to in the field as the leukemic stem cell (LSC) hypothesis: (1) a higher engraftment capacity in the bone marrow of murine recipients correlates with worse survival [58], (2) patients whose whole AML samples had a gene expression profile similar to LSCs or HSCs had worse survival and could risk stratify patients independent of known prognostic factors [59], and (3) patients with a high burden of CD34 + CD38- AML cells at diagnosis correlated with poor survival [41,42]. These findings support the notion that an optimal target would include a LSC population in the whole cell leukemic population. In this review, we focused primarily on LSC- and leukemic blast-associated cell-surface antigens for the various targeting strategies, as these are optimal targets for achieving curative therapy.

### 1.4. Optimal Targets in AML Therapy (CD33, CD123, CD13, CLL-1 and CD38)

CD33 is a 67 kDa immunoglobulin superfamily glycoprotein that is classified as a sialic acid-binding immunoglobulin-like lectin (Siglec) which has two immunoreceptor tyrosine-based inhibitor motifs (ITIMs). Phosphorylation events lead to distal activation of SHP1 and SHP2. CD33 appears on early myelomonocytic lineage-committed cells in normal hematopoiesis. It is expressed on 99% of AML blasts and LSCs.

CD123 is the interleukin (IL)-3 receptor alpha chain and is a type I transmembrane glycoprotein [60]. The beta chain (CDw131) for the IL-3 receptor is common to the IL-5 and granulocyte monocyte-colony stimulating factor (GM-CSF) receptors [61]. When CD123 is coupled to CDw131, the binding infinity for IL-3 increases dramatically, facilitating signal transduction from low concentrations of IL-3 [62]. IL-3 is important in driving myeloid differentiation and can activate STAT5. CD123 is present on ~99% of CD34 + CD38- LSCs [63] and on the majority of leukemic blasts. Importantly, CD123 is not highly expressed on normal hematopoietic stem cells [64], making it a potential therapeutic target. CD123 is expressed on committed hematopoietic progenitor cells and mediates differentiation and proliferation. CD123 is also expressed on cells of the hematopoietic system (monocytes, neutrophils, basophils, eosinophils, megakaryocytes and erythroid precursors, mast cells, macrophages, some B lymphocytes) and non-hematopoietic tissue (Leyding cells of the testis, placenta, and brain) [60].

CD13 is a zinc-dependent metalloprotease with enzymatic activity of N-terminal amino acid cleavage from peptides [65]. It is present on normal myeloid cells and is involved in several cellular functions, including adhesion, differentiation, proliferation, apoptosis, and phagocytosis, and is overexpressed on AML cells [66]. It is expressed on both blasts and LSCs. Antibodies which can bind to CD13 can not only facilitate ADCC, but also inhibit proliferation and trigger apoptosis of AML cells, but not against normal CD13-expressing blood cells [67].

C-type lectin-like receptor 1 (CLL-1) belongs to group V of the C-type lectin-like receptor family, which is calcium independent [68,69]. Based on the structure, C-type lectin and C-type-like lectin receptors are categorized into type I and type II receptor. CLL-1 is not expressed on HSCs, but is expressed on myeloid committed progenitors and on differentiated myeloid cells, such as peripheral blood monocytes, dendritic cells, and granulocytes [68]. However, it is expressed on LSCs [70], making it a potentially relevant target for antibody therapeutics.

CD38 is made up of a single chain of 300 amino acids with a molecular weight of 45 kDa and is expressed by hematopoietic and non-hematopoietic cells, including NK cells and monocytes (reviewed in [71]). Other CD38^+^ cells include smooth and striated muscle cells, renal tubules, retinal ganglion cells, and cornea (reviewed in [72]). CD38 is involved in lymphocyte signal transduction [73] and adhesion [74]. The binding of CD38 to the ligand CD31 (PECAM-1) initiates a signaling cascade that includes the phosphorylation of sequential intracellular targets and increases cytoplasmic Ca^2+^ levels, mediating different biological events, depending on the cell type (e.g., activation, proliferation, apoptosis, cytokine secretion and homing). While the absence of CD38 was used to establish classic LSC definitions [39], subsequent work demonstrated that LSCs can exist in the CD34 + CD38+ compartment of a significant number of primary AML samples [75]. Interestingly, CD38, a well-studied target for multiple myeloma [76], has been less focused on as a target for AML. This target is of potential interest for AML, given that 75% of AML samples express CD38 and there are anti-CD38 mAbs approved for multiple myeloma with established safety profiles.

### 1.5. Additional Targets in AML (WT-1, CD15, CD25, CD30, CD45)

Several other AML target candidates that have greater limitations than the previously listed antigens have been studied and are briefly summarized here. WT1 is a zinc finger transcription factor and an oncogene in AML progression and detectable in the majority of AML samples [77], but is an intracellular protein not expressed on the cell surface. However, mutations can be detected by T-cells through the presentation of mutant peptides in the context of HLA class I. This antigen presentation has been exploited by a novel BiTE construct specific for CD3 and a WT1 epitope presented in the context of HLA-A*02:01 [78]. However, this approach is limited compared with targeting tumour-associated antigens (TAAs) and may be complicated by cross-reactivity with other antigens presented with this HLA subtype [79]. CD15 is another antigen expressed on AML cells, but is only expressed on AML blasts and not LSCs, and is also present on many normal myeloid cells, making it a suboptimal target. CD25 (IL-2R alpha chain), another target selected by some investigators, is present on blasts and LSCs, but is not as widely expressed as other AML antigens, and is present on other IL-2-dependent cell types such as regulatory T-cells (Tregs), which could lead to undesirable side-effects.

CD45 is a type 1 transmembrane glycoprotein present on all hematopoietic cells [80]. It has tyrosine phosphatase activity and is involved in signal transduction with several isoforms (E3_8, CD45RO, CD45RB, CD45 RABC) derived from alternative splicing [81,82]. While CD45 is not an ideal target, as it is expressed on all hematopoietic cells, it can be used for myeloablative conditioning prior to HSCT, which is covered in the section on radio-immunotherapy.

## 2. Unconjugated Antibody Therapies

Unconjugated antibody therapy relies on important structural regions, each of which have a specific function. The basic structure of a mAb involves two identical heavy chains each with variable regions (V_H_) and two identical light chains each with variable regions (V_L_). These are joined by disulphide bonds to create the classical antibody Y structure. The fragment crystallizable (Fc) portion has regions that allow for cells of the innate immune system to bind by Fcγ receptors (FcγRs) or for complement binding. The V_L_ and V_H_ contain the complementarity determining regions which provide mAbs with a high antigen binding affinity and specificity.

**Figure 1:**
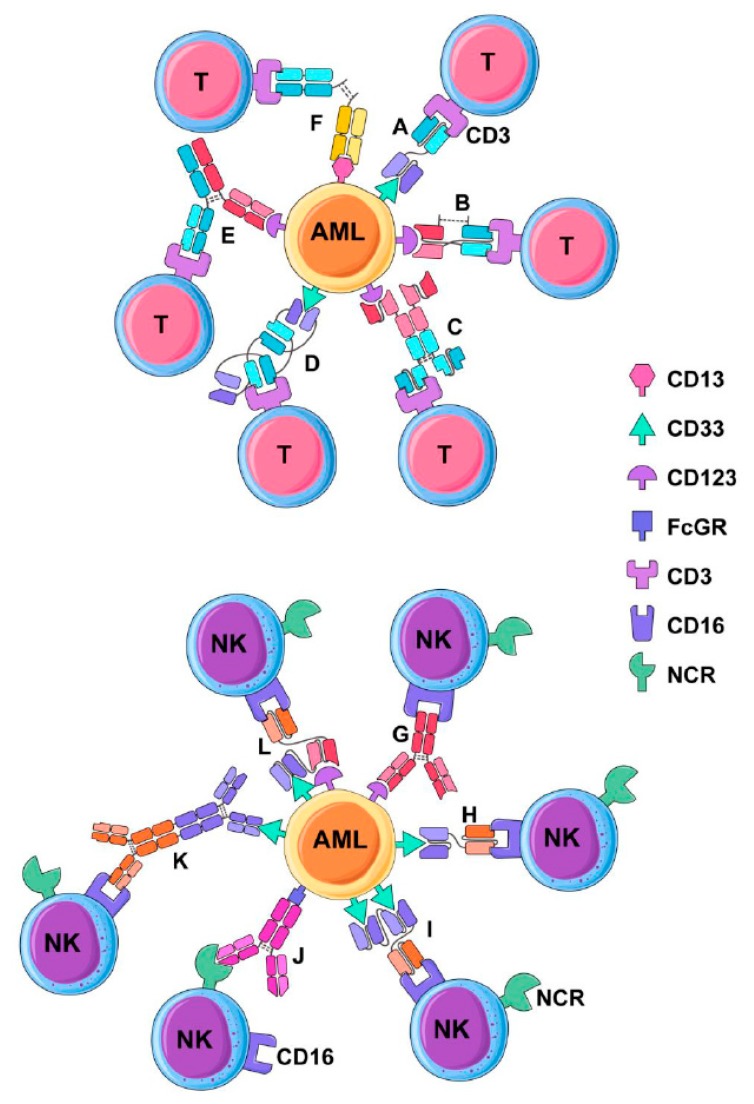
Antibody facilitated T and natural killer (NK) cell cytolysis of leukemia cells. T-cells and NK cells can be redirected to kill acute myeloid leukemia (AML) targets using a variety of antibody formats derived from the natively occurring IgG immunoglobulin molecule. Various approaches are diagrammed with key examples of each antibody format that has been developed. T-cell redirecting antibodies include (**A**) Bispecific tandem fragment variable format (BiTE, scBsTaFv), of which AMG 330 is an example (CD33 × CD3); (**B**) Dual Affinity Re-targeting Antibody (DART) (CD123 × CD3); (**C**) Bispecific single-chain Fv (scFv) immunofusion (Bif) (CD123 × CD3); (**D**) Bispecific tandem diabodies (TandAb) (AMV-564) (CD33 × CD3); (**E**) Duobody (CD123 × CD3); (**F**) Chemically conjugated Fab (CD3 × CD13). NK cell redirecting antibodies include (**G**) native IgG via antibody-dependent cell-mediated cytotoxicity (ADCC) (e.g., anti-CD123 mAb) CSL362); (**H**) Bi-specific Killer cell Engager (BiKE) (CD16 × CD33); (**I**) Tandem triple scfv (sctb); (CD33 × CD33 × CD16); **(J**) native IgG via reverse-ADCC; antibody directed against a natural cytotoxicity receptor (e.g., anti-NKp30); (**K**) Chemically conjugated antibodies (CD16 × CD33); (**L)** Tandem triple scfv (sctb); (CD33 × CD123 × CD16).

The major mechanism of action of many classical therapeutic mAbs is NK cell antibody-dependent cell-mediated cytotoxicity (ADCC), which involves exocytosis of granules containing perforin and granzymes onto target cells (Figure 1G) [83]. Following an encounter with another cell, an NK cell forms an immunological synapse (IS) [84]. If there are sufficient activation signals on the potential target cell and also a lack of inhibitory signals, a cytolytic response will be triggered. Antibody-coated targets may bind Fcγ receptor III (CD16) on NK cells and induce cytotoxicity, which can over-ride inhibitory signals. This requires cytoskeletal rearrangement and re-orientation of the granules to the IS, followed by fusion into the synapse, and subsequent contact of granule contents with the plasma membrane of the target cell. Granules contain perforin, which can create pores in the membranes of target cells following cytolytic effector degranulation [85,86]. Serine proteases termed granzymes are also contained within granules. Granzyme A was the first characterized family member in T cell granules [87,88]. Granzymes are facilitated entry by perforin, where they are able to initiate apoptosis by both caspase-dependent and independent pathways. Perforin is a 70-Kda protein that requires free calcium and neutral pH to optimally integrate into the target membrane. Granzyme B induces DNA damage through the activation of caspase activity and has been shown to partially process procaspase 3, which requires the release of other proapoptotic factors from the mitochondria to complete apoptosis [89]. By contrast, granzyme A is unable to activate caspases, but instead targets nuclear proteins directly to induce DNA single-stranded DNA breaks and fragmentation by a caspase-independent pathway [90].

Furthermore, antibodies may also bind to activating receptors on NK cells and engage FcγR on AML cells in the reverse position to classical ADCC, in a process termed reverse-ADCC (R-ADCC) (Figure 1J) first demonstrated by Saxena et al. [91]. R-ADDC can stimulate granule exocytosis with similar potency to ADCC at concentrations as low as 0.001 µg/mL by bridging an FcγR positive target with an NK cell [92]. Pre-treatment of NK cell lines with mAbs to the natural cytotoxicity receptors (NCRs), NKp30 and NKp44, can facilitate a several-fold enhancement of cytotoxicity against leukemia cell lines and primary AML blasts which express FcγRs [93]. Although no humanized anti-NKp30 antibody has been developed yet, this approach could be used to target FcγR-positive cancers, which includes approximately two thirds of the primary AML samples [94].

Antibodies can facilitate antibody-dependent phagocytosis (ADCP) by macrophage and neutrophils owing to their expression of all classes of FcγRs, in contrast to NK cells, which express only FcγRIIIA (CD16) (reviewed in [95]). Antibody-coated cancer cells may become engulfed by macrophages or neutrophils, leading to their destruction and also to antigen presentation that could result in an adaptive anti-tumour immune response. Elotuzumab (anti-SLAMF7) has been approved for treatment of multiple myeloma and has demonstrated ADCP as a mechanism [96] in addition to ADDC and NK-cell activation.

Antibodies can also mediate cytotoxicity against cancer cells by complement dependent cytotoxicity (CDC) via the classical pathway (reviewed in [97]). Briefly, CDC involves initial binding of C1 via its C1q subcomponent to two adjacent cell-bound antibodies, leading to the recruitment of other components (C4, C2) and ultimately forming a C3 and C5 convertase. This leads to another cascade involving components C5-C9 with the final formation of the membrane attack complex, which results in pore formation in the target cell, and ultimately, osmotic lysis.

### 2.1. CSL360/CSL362 (Talacotuzumab)

The murine anti-human CD123 mAb 7G3 has been modified into two versions: chimeric CSL360 and humanized CSL362 (talcotuzumab). CSL360 has the variable region of 7G3 and is fused with the backbone of a human IgG1 through genetic engineering. Unfortunately, in a phase I clinical trial of 40 relapsed and refractory AML patients, CSL360 was unable to provide therapeutic benefit in all but two cases [98]. As a consequence, CSL360 was not pursued for further clinical development. A second-generation version of this antibody, CSL362, was Fc optimized to bind CD16A on NK cells with better affinity, as well as affinity matured to better bind to CD123 by its variable region (Figure 1G). CSL362 was tested in a Phase 1 clinical trial of AML patients in first or second CR as consolidation therapy which, at interim report, had 25 patients and was generally well tolerated, with three severe adverse events reported and no deaths from toxicity [99]. Ten patients maintained a CR for greater than or equal to 6 months (median 34 weeks) and of six MRD-positive patients, three converted to MRD-negative. While a further Phase II study of talocotuzumab and decitabine for AML (NCT02472145) was completed by Janssen, this trial did not meet the endpoint criterion to justify the further development of CSL362. However, these clinical results indicate that targeting CD123 has some anti-leukemic effect, when the mAb is engineered to provide more potent ADCC activation.

### 2.2. Lintuzimab and Bl 835858

Lintuzumab (SGN-33, HuM195) is an unconjugated anti-CD33 mAb which has been tested in several clinical trials for AML (NCT00002609, NCT00002800, NCT00006084, NCT00016159, NCT00283114, NCT00502112, NCT00528333, and NCT00997243) in combination with standard induction chemotherapy and a maintenance monotherapy in R/R AML (reviewed in [100]). Both a phase 2b randomized trial [101] and a phase 3 randomized trial [102] of lintuzumab did not demonstrate survival benefit and the agent was not pursued further but has been applied for the delivery of radionuclides and described in a subsequent section. Another unconjugated anti-CD33 mAb, BI 836858, is Fc optimized through engineering, leading to improved NK cell-mediated ADCC relative to native antibody Fc [103]. Several clinical trials of BI 836,858 are currently underway for R/R AML (NCT02632721 NCT01690624) and AML relapsing post HSCT (NCT03207191).

### 2.3. Daratumumab (Darzalex), Isatuximab

Daratumumab is a fully human IgG1 kappa mAb that targets CD38 and was generated using the HuMAb platform with human antibody transgenic mice [104]. Daratumumab was originally evaluated in and approved for use in multiple myeloma (MM) in 2015 [105]. Daratumumab has been tested against primary AML targets in vitro, demonstrating apoptosis induction, ADCC and CDC as mechanisms of cytotoxicity, as well as being shown to reduce leukemic burden in the spleen and peripheral blood, but not in bone marrow in primary AML xenograft models [106]. Its mechanism of action includes (CDC), ADCC [107] and (ADCP). Investigators at MD Anderson Cancer Center are currently evaluating the efficacy of daratumumab as a stand-alone treatment for R/R AML (NCT 03067571), while Ohio State University is evaluating its effectiveness in combination with donor leukocyte infusions (DLI) for AML patients who have relapsed after allogeneic HSCT (NCT 03537599).

Another anti-CD38 antibody, isatuximab, has also been tested in MM, NHL, and CLL patients in a phase 1 trial [108] and a phase 3 trial in patients with MM, which showed improvement in progression-free survival. One advantage of isatuximab versus daratumumab is the need for less frequent dosing, though no head-to-head comparison of efficacy has been performed. A study of isatumximab recently opened a phase I/II trial for pediatric patients with R/R AML and ALL for use with combination chemotherapy (NCT 03860844).

## 3. Multivalent Antibody Therapies

Multivalent antibodies with, bi-, tri- and quadri-specific binding domains are engineered constructs which combine specificities of two or more antibodies into one molecular product that is designed to bind to both a TAA and an activating receptor on the effector cells, typically a T cell or natural-killer (NK) cell. There are several different structural variants of bispecific antibodies, which, in turn, can be utilized to target various combinations of effector and tumour targets antigens [109]. The first FDA-approved dual-binding antibody was blinatumomab, a bispecific T-cell engager (BiTE) developed by Amgen with specificities for CD3 and CD19 for treatment of acute lymphoblastic leukemia (ALL). BiTE format antibodies (tandem di-scFv) are engineered products involving combining the V_L_ and V_H_ domains of a monoclonal antibody into a single chain fragment variable (scFv) specific to an activating receptor (e.g., CD3) and further linked to the scFv of an antibody specific to a target antigen (e.g., CD19). The CD19 × CD3 BiTE was able to induce remissions in relapsed and refractory ALL that had failed other therapies [110,111] and was recently approved by the FDA for ALL patients with minimal residual disease (MRD) [112]. Because of the low molecular weight of blinatumomab, it is rapidly cleared by the kidneys and excreted through the urine, requiring administration by continuous infusion for up to several weeks [110]. Hence, for clinical use, there are challenges in cost and practical applications. Nonetheless, this technology is powerful, as it can be used as a general approach to redirect T-cells toward any antigen of interest. It can also be applied to engineered antibody fragments with different formats than the BiTE, such as DART and Duobody, also increasing valency, such as with tri-specific antibodies.

In general, most bi- and tri-specific engineered antibodies lack an Fc region and therefore, do not have the ability to mediate CDC and ADCC. Moreover, the engagement of NK cells to targets by bi- or tri-specific antibodies can result in immediate degranulation, owing to their preformed granules [113,114], whereas only a subset of primed T-cells can optimally be redirected to mediated cytotoxicity against against cancer-cell targets [115]. Only three bi-specific antibodies have been entered into clinical trials for AML, which are registered on ClinicalTrials.gov (Table 1) and are discussed in more detail with other bi-, tri- and quadri-specific formats in the pre-clinical stage of development.

### 3.1. Bispecific Tandem Fragment Variable Format (BiTE, scBsTaFv)

Given the success of blinatumomab for ALL, Amgen subsequently developed AMG 330, which is a CD33 × CD3 specific BiTE for treatment of AML (Figure 1A). The CD33 × CD3 BiTE can facilitate T-cell activation, expansion and in vitro lysis of primary AML cells [116,117]. Furthermore, AMG 330, in combination with infusions of activated human T cells, could suppress the growth of AML cells in a MOLM-13 cell line xenograft model, leading to improved survival [118]. Furthermore, AMG 330 could facilitate the cytotoxicity of both autologous and healthy donor allogenic T-cells against primary AML [119]. Recently, preliminary data for AMG330 was presented at the American Society for Hematology (ASH) meeting for patients with relapsed and refractory AML (NCT02520427) [120]. This was a phase 1 dose escalation study with 35 patients (enrolment to 40 in progress) to evaluate the safety, pharmacokinetics and tolerance. Four patients (10%) achieved a CR or a CR with incomplete blood count recovery treated with 120 or 240 ug/day. However, these CRs were not sustained beyond one cycle of treatment and the majority of patients discontinued treatment because of disease progression. Adverse events included cytokine release syndrome (CRS), febrile neutropenia, pneumonia, leukopenia, pyrexia, thrombocytopenia and subdural hematoma. Although this study established a tolerable dose, there was a low frequency of remission induction and short durations of remission in the four responders.

Another bispecific antibody format termed single-chain bispecific tandem fragment variable (scBsTaFv) provides an alternative to tandem scFv fragments in the BiTE format. This approach attaches the V_H_ of an anti-CD3 mAb to the V_L_ of an anti-CD33 mAb with the two tandem scFv joined by a glycine-serine linker. Further optimization of this format was resolved by rearranging the variable regions. A CD33 × CD3 scBsTaFv construct was able to facilitate the cytotoxicity of allogeneic mononuclear cells (PBMC) against CD33-expressing target cells [121]. This CD33 × CD3 scBsTaFv bispecific antibody was further humanized, reducing the probability of the patient mounting an immune response against the agent and was effective in picomolar concentrations, was independent of CD33 antigen density and did not redirect cytotoxicity to HSCs, as measured by a clonogenic assay [122]. An additional modification was to add 4-1BB ligand (4-1BBL), a co-stimulatory molecule which further enhanced T cell cytotoxicity against AML cells, against CD33_low_ targets, relative to the parental construct [123].

### 3.2. Dual-Affinity Retargeting (DART)

Dual-affinity retargeting (DART) molecules are generated from a V_H_ and V_L_ taken from two distinct mAbs in a format distinct from BiTE to target two distinct antigens (Figure 1B). Specifically, a CD123 × CD3 DART was developed by Macrogenics (flotetuzumab), which can redirect T cells to target and kill AML cells, as well as stimulate T-cell proliferation [124]. The CD123 × CD3 DART was able to suppress leukemia progression in a CD123 + GFP + CBRLuc K562 murine xenograft model as measured by bioluminescence. The preliminary results of a phase 1 clinical trial of flotetuzumab demonstrated that it had anti-leukemic activity in 57% of a cohort of 45 patients with R/R AML/MDS (89% AML). Toxicity was reported and included infusion-related fever, chills, tachycardia, and hypotension, which were not severe. Importantly, the overall response rate (ORR) was 43%. Thus, the preliminary results are encouraging for DART as a potential effective targeted therapeutic for R/R AML.

### 3.3. Bispecific scFv Immunofusion or BIf

A novel and interesting bi-specific antibody format is the Bispecific scFv Immunofusion (BIf) with an scFv fused at the N-terminus of human IgG1 hinge region with a second scFv at the C-terminus (Figure 1C). The first described BIf was an CD123 × CD3 construct which would form pairs as a homodimer with a tumour target binding Kd of 1.0 × 10^−10^ molar which is superior to other bispecific antibody formats and can facilitate T-cell mediated cytotoxicity in vitro at low effector:target ratios [125]. Due to an intact Fc, it has a longer half-life than the lower molecular weight classical BiTE molecules and, hence, a longer therapeutic exposure to AML cells.

### 3.4. Bispecific Tandem Diabodies (TandAb)

Bispecific tandem diabodies (TandAb), also termed bispecific tetravalent antibodies, have a unique structure with two homologous immunoglobulin chains running counter to each other (V_H_A-V_L_B-V_H_B-V_L_A)_2_ (Figure 1D) [126]. A CD30 × CD3 TandAb (AFM13) is being tested in clinical trials for cutaneous lymphoma (NCT 03192202—recruiting), R/R Hodgkin lymphoma (NCT 02321592 (recruiting and NCT 02665650—completed). A CD33 × CD3 TandAb has been generated (AMV-564) which, in the presence of T cells, can mediate dose-dependent cytotoxicity against primary AML targets from newly diagnosed and refractory or relapsed patients in vitro. AMV-564 had efficacy in treating a murine AML xenograft model [127]. A trial of AMV-564 has been initiated for R/R AML (NCT 03144245) and is sponsored by this platform’s developer, Affimed Therapeutics Inc. ( Heidelberg, Germany).

### 3.5. Chemically Conjugated Bispecific Antibodies

An early approach to dual target antigens was to chemically conjugate two different antibodies. A chemical conjugate of anti-CD16 and anti-CD33 monoclonal antibodies was developed which could redirect the cytotoxicity of NK cells toward AML blasts (Figure 1K) [128]. Another variant of this approach was to develop a bispecific (Fab’)_2_ fragment derived from two different antibodies [129,130]. Specifically, antibodies underwent cleavage and separation of (Fab’)_2_ fragments with dithiothreitol to create Fab’-SH fragments which could be recombined using a thiol-disulfide interchain reagent, ultimately producing a bispecific hybrid F(ab’)_2_. This approach was used to combine an anti-CD3 (OKT3) and anti-CD13 mAb (Figure 1F) [131]. These anti-CD3 and anti-CD13 Fab’ fragments were mixed and reduced to form a bispecific F(ab’)_2_. This CD13 × CD3 Fab was able to enhance lysis of AML blasts by PMBCs. Also, this CD13 × CD3 antibody construct had some inhibition of AML colony-forming units (CFU), and had a lesser effect on granulocyte/macrophage CFU from normal bone marrow [131].

### 3.6. Bispecific Full-Length Antibodies (Duobody and Biclonics)

Genmab developed the DuoBody platform to develop bispecific human IgG1 antibodies. Two mAbs of different specificity, each containing single matched mutations in the third constant (C_H_3) domain, were produced using mammalian recombinant cell lines and are then in a bispecific antibody. A CD123 × CD3 duobody was developed from an IgG4 backbone with a silenced Fc function and termed JNA-63709178 (Figure 1E) [132]. A phase 1 trial of a CD123 × CD3 Duobody (JNJ-63709178) sponsored by Janssen is in active recruitment of R/R AML patients (NCT 02715011). A full-length bispecific CLL-1 × CD3 antibody was developed by Merus using their proprietary Biclonics platform with preclinical activity demonstrated against AML [133], with an ongoing Phase 1/2 clinical trial ongoing for R/R AML in adults and newly diagnosed elderly patients with complex cytogenetics (NCT 03038230).

### 3.7. BiKEs and TriKEs

A CD33 × CD16 bispecific scfv was designed to activate NK cells and redirect them to lyse CD33+ AML targets and also secrete cytokines (IFNγ and TNFα) [134]. This construct is structurally similar to BiTE and was termed a bispecific killer-cell engager (BiKE) (Figure 1H). The observation that CD16 can be shed from NK cells by cleavage with ADAM17 limited this approach, but this was overcome with an ADAM17 inhibitor that could enhance NK cell cytotoxicity and cytokine secretion in the presence of the CD33 × CD16 BiKE. The BiKE platform was also functional using NK cells from patients with myelodysplastic syndrome, facilitating cytotoxicity against both AML targets and myeloid-derived suppressor cells [135].

Further modification of BiKEs was carried out to incorporate a third functional domain, specifically, to incorporate the IL-15 cytokine, and accordingly named a trispecific killer cell engager (TriKE) [136]. This approach facilitated additional expansion and activation capacity of NK cells, and when compared to the BiKE, yielded superior anti-leukemic results in mouse models of human AML. Three scFv components can be inserted to create a (scFv)_3_ construct termed a single-chain Fv triplebody (sctb). One such construct linked two anti-CD33 scFv fragments to a single anti-CD16 scFv and was compared with a bispecific format (bsscFv) that consisted of only a single anti-CD33 scFv (Figure 1I). The CD33 × CD33 × CD16 sctb had a greater binding affinity for CD33 compared to the affinity obtained with the CD33 × CD16 bsscFv. More importantly, the sctb had increased by ≥2-logs the NK cell cytotoxic potency against AML cells relative to bsscFv [137]. Another approach using the triple scFv involves targeting two different antigens on a single target cell (Figure 1L). Using this approach, a CD33 × CD123 × CD16 was developed and shown to facilitate superior leukemic cell killing by NK cells relative to the dual targeting of the same antigen (CD123) [138]. A clinical trial of TriKE therapy is underway for patients with CD33 + R/R AML (NC T03214666).

## 4. Toxin-Conjugated Antibody Therapy for AML

Conceptually, the combination of highly potent anti-neoplastic agents and targeted antibodies in a single antibody-drug conjugate is not a recent development [139]. The principle of attaching a targeted antibody to a cytotoxic drug or radioactive isotope (referred to as ‘payload’ or ‘warhead’) through covalent linkage has led to a number of antibody-drug conjugates (ADCs) approved for use in the management of hematological malignancies. Although several warheads are being tested in cancer, the warheads that will be described have been used in AML.

Structurally, there are three components of equal importance in the design of an ADC, namely the mAb itself, the cytotoxic agent, and the conjugation linker [140]. The mAbs can be human, humanized, or chimeric, and may be engineered to target the antigen of choice with high specificity. For optimal drug delivery, the linker must bind with sufficient integrity to prevent premature de-conjugation, yet must release the drug once the antibody has bound to the target [141]. Linkers may be cleavable or non-cleavable. Typically, ADCs are reliant on efficient degradation in lysosomes to release the payload inside the target tumour cell [140]. Each approach is associated with its unique advantages and drawbacks and should be engineered with the target cell and the payload in mind. Finally, the drug itself must be sufficiently potent to ensure tumour killing with minimal off-target toxicity.

Calicheamicin is an anti-tumour antibiotic synthesized from *Micromonospora echinospora* that induces double-stranded DNA breaks, leading to cell death [142]. This is used in conjugation with antibodies targeting CD33 (gemtuzumab ozogamicin/Mylotarg) in AML, or CD22 (inotuzumab ozogamicin) in B-cell acute lymphoblastic leukemia (ALL). Monomethyl auristatin E (MMAE) is conjugated to an anti-CD30 antibody in brentuximab vedotin, an ADC that is FDA-approved for Hodgkin lymphoma [143]. Most recently, an investigational agent was developed using a pyrrolobenzodiazepine (PBD) dimer to induce DNA damage in tumour cells [144]. Vadastuximab talirine (SGN-CD33A) is a third generation ADC construct whereby an anti-CD33 antibody is conjugated to two molecules of a pyrrolobenzodiazepine (PBD) dimer via a maleimidocaproyl valine-alanine dipeptide connecting segment [145]. The PBD dimer is released after protease cleavage and induces DNA cross-linking, leading to target-cell apoptosis [146]. Several ADCs for AML have been tested in publish clinical trials and are discussed below, while other novel ADC trials for AML are ongoing.

### 4.1. Gemtuzumab Ozogamicin (GO)

The first clinically viable ADC to be approved in hematological malignancies was gemtuzumab ozogamicin (GO; Mylotarg), which targets CD33 [147]. In phase III studies of GO as monotherapy in patients over the age of 60 with relapsed AML, an overall response rate of 30% was reported. Based on these data, GO received accelerated FDA approval in 2000 [148]. However, a subsequent multicenter phase 3 randomized clinical trial comparing GO 6 mg/m^2^ on day 4 of a daunorubicin and cytarabine induction chemotherapy protocol failed to demonstrate differences in survival. In fact, the patients receiving GO had a higher rate of mortality during induction due to Veno-Occlusive Disease (VOD) (5.5% death rate in the combination arm versus 1.4% in the chemotherapy alone arm) [149]. As a result, the drug was voluntarily withdrawn from the market in 2010. However, subsequent randomized trials evaluating lower doses of GO in combination with chemotherapy demonstrated improved overall survival without increased toxicities such as VOD. For example, The MRC AML15 trial combined GO at a dose of 3 mg/m^2^ on day 1 of conventional induction chemotherapy in 1113 patients with previously untreated AML and reported a significant survival benefit without increased toxicity in younger patients with favorable cytogenetics, particularly core binding factor leukemias [150]. Another trial of a similar dosage regimen in older patients showed no difference in CR rates but significantly improved the three-year OS and relapse-free survival (RFS) with no appreciable increase in toxicity [151]. This demonstrated that lower doses of GO are effective in AML. As a result, GO was reapproved by the FDA for the treatment of newly-diagnosed CD33-positive acute myeloid leukemia (AML) in adults and for treatment of relapsed or refractory CD33-positive AML in adults and in pediatric patients 2 years and older.

### 4.2. Vadastuximab Talirine (SGN33A) and IMGN779

Other ADCs that target CD33 have been developed and are being actively investigated for AML therapy. Early in vitro experiments and animal studies showed that SGN33A was active even in multi-drug resistant and p53 mutated AML cell lines [152]. A phase 1 trial of SGN-CD33A in 27 older (median age 74 years) treatment naïve AML patients reported responses in 54%, with 14 patients achieving CR/CRi and five achieving a morphological leukemia-free state [153]. MRD negativity by flow cytometry was noted in six of 13 patients for whom data were available. VOD was not reported with this drug. Current trials are focused on evaluating SGN-CD33A in combination with standard induction chemotherapy. For example, a trial of SGN-CD33A in combination with decitabine or azacitidine in 24 patients with AML unfit or unwilling for conventional chemotherapy had a response rate (CR + CRi) of 73% amongst 49 evaluable patients [154]. In addition, 47% of the responding patients achieved MRD negativity by flow cytometry. Noteworthily, the combinations were well tolerated, with a 30-day mortality rate of 2%. Another CD33-directed antibody-drug conjugate IMGN779 in which the mAb is bound to a novel alkylating agent DGN462 was active in preclinical models [155] and a phase 1 clinical trial is currently underway in R/R AML (NCT 02674763).

### 4.3. Current Clinical Trials

There are many current clinical trials evaluating ADCs for AML which are registered on ClinicalTrials.gov (Table 2). GO is under investigation as a single agent for R/R AML in a phase 2 trial (NCT 03374332). Given that treatment of R/R AML is challenging and would not address its role in treating minimal residual disease (MRD), a phase 2 trial of GO is being examined after standard induction chemotherapy in patients achieving a CR who remain MRD + (NCT 03737955). Finally, another phase 1/2 study is aiming to evaluate the efficacy of GO in combination with chemotherapy in the up-front setting (NCT 03531918). Another CD33-directed antibody-drug conjugate IMGN779, in which the mAb is bound to a novel alkylating agent DGN462, was active in preclinical models [155], and a phase 1 clinical trial of this agent is currently underway in R/R AML (NCT 02674763). Other currently active clinical trials are examining the role of ADCs targeting CD30, CD123, CD71, and *FLT*3 in patients with AML and other hematological malignancies expressing these antigens.

## 5. Radioimmunotherapy of AML

Radioimmunotherapy (RIT) for R/R AML is a scientifically sound approach that is translatable to clinical practice and can improve treatment outcome in patients. RIT utilizes mAbs labeled with radionuclides, providing continuous ionizing particle-based radiation exposure to cells expressing target antigens. RIT for AML has been investigated for the past three decades, demonstrating its ability to kill leukemic cells and deliver radiation specifically to sites harboring AML disease.

Although clinical testing is in place, important aspects such as target antigens on AML (or also on normal hematopoietic) cells and the properties of the payload radionuclides are still being optimized. Therefore, in this section, we put into context how RIT works and demonstrated the unique properties of select radionuclides and what has been learned from several clinical trials. Here, we identified clinical trials that are currently recruiting patients or are in preparation.

Due to exquisite affinity and specificity for target antigens preferentially expressed on diseased cells by mAbs and because radiolabeled mAbs have an increasingly well-understood pharmacokinetic profile, they target tumours with high target-to-nonspecific organ ratios [156]. Hence, radiolabeled mAbs are able to deliver ionizing radiation to disease sites more specifically than traditional total body irradiation (TBI). Ionizing radiation is then delivered with increased precision to AML cells and induces cell-death by two main mechanisms—apoptosis and necrosis—as a result of unrepaired DNA strand breaks [157]. Although RIT efficiency depends on many additional factors, this is beyond the scope of this summary and was reviewed in Desouky et al. [158].

A key RIT property is the type of radionuclide that is attached to the mAb. The nature of radiation emitted from radioisotopes that radioactively decay via α-particle or β-particle emission is different (Table 3). For example, the most relevant radionuclides for AML ^225^Ac and ^211^At decay by the emission of α-particles, whereas ^131^I and ^90^Y decay by the emission of β-particles. These radionuclides have different energies and another unique important property called linear energy transfer (LET). LET is the amount of energy that an ionizing particle transfers to material traversed per unit distance. Typically, α-particles have a range in tissue of 50–80 μm, which results in an LET of 50–230 keV/μm (Table 1). In contrast, β-particles have a considerably reduced LET of 0.1–1 keV/µm as they have a range of 0.5–12 mm in tissue. This signifies that α-particles provide an increased relative biological effectiveness (RBE) relative to β-particles. RBE is a measure of the extent of damage (e.g., DNA double strand breaks) to the cell an emitted particle produces. The RBE for α-particles is 3–7-fold increased relative to β-particles [159].

In general, ^131^I and ^90^Y have been employed in >95% of clinical RIT trials and represent the current standard to which all other radionuclides are compared [160]. Both isotopes have distinct favorable properties. ^131^I emits a second ionizing particle, the γ-ray and can thus be utilized for both imaging and therapy. However, ^131^I-mAbs degrades rapidly if the receptor internalizes upon mAb binding. This results in the release of ^131^I-tyrosine in the bloodstream. In addition, the γ-rays emitted by ^131^I increases the absorbed radiation dose to tissues and pose a risk to family members and healthcare professionals and, hence, require patient isolation. Both of these properties increase the absorbed radiation to healthy organs. Alternatively, ^90^Y is an almost exclusive β-particle emitter (Table 3). Since the travel distance of β-particles is short compared to γ-rays and does not leave the patient’s body, ^90^Y is safer and more practical to work with for healthcare professionals. ^90^Y also emits β-particles that are more energetic by 5-fold compared to the β-particles emitted by ^131^I. In addition, if ^90^Y-mAbs are internalized, the isotope is retained in the cell and is not released into the bloodstream. With either case, it is vital to have knowledge that the radionuclide targets tissue harboring the leukemic cells several fold higher than the liver, kidney, and lung—the dose limiting normal tissues. Since ^90^Y cannot be imaged, dosimetry relies on utilizing another isotope labeled on the mAb, typically 111-indium (^111^In).

**Figure 2:**
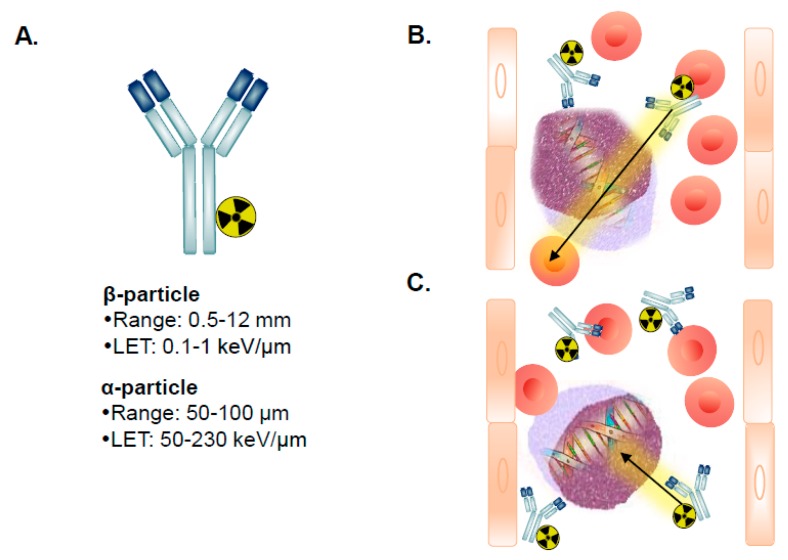
Radioimmunotherapy for acute myeloid leukemia. Illustration of (**A**) a mAb radiolabeled with either a (**B**) β-particle or (**C**) α-particle-emitting radionuclide and the track of the particles perfusing the bone marrow to target AML cells. Note that the path length of β-particles is greater than for α-particles leading to β-particle-based RIT used primarily in preparative regimens to myeloablate the bone marrow prior to hematopoietic cell transplantation.

A caveat for β-particle-based RIT is that the emitted β-particles often overshoot single AML cells and ablate surrounding normal hematopoietic cells residing in the bone marrow [161] (Figure 2A,B). This often results in dose-limiting toxicity (DLT) to the bone marrow, as normal hematopoietic cells are also highly sensitive to irradiation [162]. In contrast, the short range of emitted α-particles in theory makes them ideal for eradicating individual leukemic cells (Figure 2A,C). However, the energy of α-particles is several-fold higher than β-particles and thus, can also cause unwanted toxicities.

Although this is not a focus in this brief summary, short-ranged and high LET Auger electrons may provide an approach to eradicate AML cells. Recent preclinical studies have shown that the Auger-emitter ^111^In labeled to mAbs targeting CD33 and CD123 and conjugated to short peptides harboring a nuclear localization signal sequence could kill AML cell lines and primary AML cell engrafted into mice. In addition, single-photon emission computed tomography (SPECT) imaging could be used as a companion to evaluate the targeting of AML cells in the bone marrow and extramedullary sites in these preclinical models to the γ-ray emissions by ^111^In [163,164,165,166].

### 5.1. β-Particle RIT for AML

Because of the properties of β-particles and the need to overcome the limitation of normal organ tolerance with current preparative regimens HSCT, The Fred Hutchinson Cancer Research Center (FHCRC) made an important breakthrough for developing an effective niche for RIT utilization. As detailed in a review by Gyurkocza and Sandmaier, successful HCT relies on the effectiveness of the preparative or conditioning regimen administered to patients prior to transplantation [167]. The goals of the regimen are to provide sufficient immunoablation to prevent the graft rejection of the donor cells and to reduce the leukemic burden [167].

In general, myeloablative regimens consist of alkylating agents with or without TBI that ablate marrow hematopoiesis to the point where autologous hematologic recovery does not occur. The greater part of regimens combines 12- to 16-Gy TBI, typically fractionated, with chemotherapy, and includes cyclophosphamide, cytarabine, etoposide, melphelan, and busulphan [167]. These chemotherapeutic agents have shown the ability to simultaneously exert cytotoxic and cytostatic effects on leukemic cells and to suppress the patient’s own immune system to reduce graft rejection. While increased doses of TBI reduce the risk of AML relapse, this also increases treatment-related mortality (TRM), often due to toxicity in the gastrointestinal system, the liver, and the lungs. Hence, HSCT is not an option for many older and medically infirm patients. In addition, TBI-induced malignancies and development impairment in children are significant concerns.

As an alternative, non-myeloablative regimens have been developed for patients with AML that provide more favorable toxicity profiles yet are sufficiently immunosuppressive. There is a considerable range in intensity of these regimens. However, the approach developed at FHCRC is relevant since most RIT trials have occurred there. In particular, in older patients, conventional conditioning regimens prior to HCT often leads to a high TRM [168]. A low-dose 2-Gy TBI-based preparative regimen is administered to patients at FHCRC with the addition of 90 mg/m^2^ of fludarabine to prevent graft rejection and increase pre-transplantation host T-cell immunosuppression [169]. Cyclosporine and mycophenolate mofetil is also administered to increase immunosuppression.

Although high-intensity TBI doses reduce relapse rates, this is offset by increased conditioning regimen-related death [170,171]. Conversely, reducing the TBI allows for increased utilization of HCT for patients with AML, however AML relapse rates increase [172,173]. Hence, there is a dilemma to find an ideal regimen that allows for low toxicity yet potent myeloablation/myelosuppression before transplantation that improves patient survival. Thus, targeted radiation is an alternative.

The mAb BC8 is specific for CD45 and radiolabeled with ^131^I was developed as means to myeloablate since the antigen is expressed on the surface of almost all hematopoietic cells, except mature red blood cells and platelets and is also present on AML blasts [174]. CD45 is an attractive target because it is expressed by most AML samples at relatively high levels (~200,000 receptors per cell), and the antigen does not internalize, which is important for ^131^I. Because CD45 is expressed on both normal and leukemic cells, it can be used to target the bone marrow. The strategy is to reduce the radiation dose and, hence, the overall toxicity to the patient by delivering radiation specifically to sites harboring leukemia cells.

An early clinical trial evaluating RIT for AML combined escalating doses of radiation delivered by the ^131^I-BC8 with cyclophosphamide and 12-Gy TBI in patients with advanced AML [175]. Patients received a “tracer” infusion of the radiolabeled mAb weeks before HCT to allow for the mAb to penetrate the hematopoietic tissues harboring the leukemic cells and for sufficient washout from the blood and normal organs. At ~72 h post-injection, patients were imaged with a focus on region-of-interests such as the bone marrow (acetabulum and sacrum), spleen, liver, lungs, kidneys, and spleen. The study showed that 84% of patients contained radiation in the bone marrow and spleen 2.3- and 4.8-fold higher than the liver, the normal organ receiving the highest dose due to normal mAb metabolism. The ratios of radiation delivered to the bone marrow and spleen relative to the lung and kidneys were even greater than the liver. More importantly, this procedure could be safely performed with a conventional HCT preparative regimen.

The effectiveness of targeting radiation to specific hematopoietic/AML sites was then expanded to patients with AML in a first remission with a human leukocyte antigens (HLA)-identical family donor [176]. In addition to ^131^I-BC8, patients underwent a preparative regimen consisting of busulfan and cyclophosphamide without TBI. The three-year survival among the patients in this study was 63% and the TRM was 21%. In patients with unfavorable cytogenetics, the percentages of survival and TRM were 26% and 27%, respectively. This study demonstrated that ^131^I-BC8 could be used to intensify the amount of radiation delivered to leukemic cells to a greater extent than that delivered to normal organs, which provided increased survival benefit without excessive TRM. As a result, the effectiveness of ^131^I-BC8 is now being evaluated to validate its use for AML as part of preparative conditioning prior to HSCT. Distinct evaluations are currently active, such as testing ^131^I-BC8 in combination with fludarabine and 2-Gy TBI for allogeneic HCT, specifically in patients ≥50 years old (NCT 00008177). This trial reported outcomes in 58 patients with R/R-AML or myelodysplastic syndrome (MDS) [177]. Treatment resulted in the MTD of ^131^I-BC8 delivered to the liver was 24-Gy. At day 28 post-transplantation, all the patients had a complete remission and donor-cell engraftment. Seven patients ultimately succumbed to TRM by day 100. The one-year survival estimate was 41%. Another phase 1 trial is evaluating ^131^I-BC8 with fludarabine and 2-Gy TBI in 15 R/R-AML patients that were ≥16–50 years of age (NCT 00119366) [178]. This study capped the maximum dose delivered to the liver at 43-Gy as was carried out in the trial with older AML patients out of concern for causing stromal damage and marrow failure. In these younger patients, no cases of DLTs or TRM were observed. At ~12 months since treatment, 11 of the 15 patients survived and at five years, there were six surviving patients. This study suggested that increased doses from ^131^I-BC8 are tolerable and provide even more survival benefit to younger adults with R/R AML. The results from these completed and active trials show the feasibility of using ^131^I-BC8 to enhance the efficacy of allogeneic HCT in both young and older patients in a reduced conditioning setting.

One area in need of progress to extend RIT is for patients who do not have closely HLA-matched allogeneic donors and instead are reliant on haploidentical donor engraftment, especially patients from ethnic minority groups. Orozco et al. demonstrated that ^90^Y-anti-CD45 RIT and cyclophosphamide without TBI or fludarabine before haploidentical HCT resulted in a high rate of engraftment, which remained stable for six months after transplantation in a murine mouse model [179]. An ongoing phase 2 trial is evaluating the use of ^131^I-BC8 in R/R AML patients receiving haploidentical donor HCT (NCT00589316). Thus far, six of eight patients have been reported to achieve complete remission by day 28 post-HSCT and all patient had 100% donor chimerism [180].

As previously described, a significant barrier to expanding ^131^I-BC8 as a mainstream approach to treat patients with AML is that many Nuclear Medicine Departments have difficulty properly handling large quantities of ^131^I. Thus, a phase 1 study was undertaken to establish the safety, feasibility and optimized dose of ^90^Y-BC8 in patients undergoing reduced-intensity regimen before allogeneic HCT (NCT01300572). Favorable outcome measures were reported with ^90^Y-BC8 for multiple myeloma and lymphoma [181,182]. A previous Phase 1 clinical trial studying ^90^Y conjugated to rat mAb YAML568 that recognizes CD45 was evaluated in eight patients [183]. No significant administration-related side effects were observed. However, in order for ^90^Y-YAML568 to deliver preferentially increased radiation doses to the bone marrow and spleen, the patients were required to be preloaded with cold antibody. This indicates that patients may have produced an anti-YAML immune response that causes clearance of the mAb. Patient outcome was not reported.

^90^Y has also been conjugated to the mAb lintuzumab that targets CD33. CD33 is exclusively expressed on myeloid cells and lymphocytes and not on all hematopoietic cells, such as CD45. Importantly, as CD33 is frequently expressed on AML cells it is an attractive target for RIT. Lintuzumab is a humanized mAb and binds CD33 with a very high affinity, as previously discussed. Two completed trials (NCT 00002890 and NCT 00006040) have investigated the MTD of ^90^Y-BC8 in patients with R/R AML with and without HCT, respectively. No findings have yet been published.

### 5.2. α-Particle RIT for AML

The relative increased energy of α-particles, relative to β-particles, coupled with a range of only a few cell diameters and high LET in theory makes this RIT approach ideal for the treatment of small-volume disease or MRD. Hence, there has been high clinical activity to test α-particle-based RIT in AML. A Phase 1 study from the 1990s evaluating ^213^Bi-lintuzumab administered at escalating doses in patients with R/R AML revealed that the absorbed dose was preferential to the bone marrow and spleen and was calculated to be 1000-fold higher than for β-particle-emitting radionuclides [184,185]. A subsequent phase I/II trial of ^213^Bi-lintuzumab produced remissions in patients after partial leukemic cell reductions were achieved due to patients receiving cytarabine (NCT 00014495). The MTD of ^213^Bi-lintuzumab in combination with cytarabine was 37 MBq/kg. Partial or complete remissions were observed in 34% of patients. However, no responses were achieved for patients receiving reduced doses of ^213^Bi-lintuzumab. These studies provided proof-of-concept for α-particle RIT, however the 45.6 min half-life of ^213^Bi and the requirement of an on-site generator makes its application challenging.

A phase 1 trial of ^225^Ac-lintuzumab was evaluated in 18 patients with R/R AML (NCT 00672165). Patients tolerated doses of ^225^Ac-lintuzumab up to 111 kBq/kg and peripheral AML blasts were eliminated in 63% of patients at doses starting at 37 kBq/kg. Importantly, extramedullary toxicities were limited to transient grade-3 liver-function abnormalities in three patients. No evidence of radiation-induced nephrotoxicity was observed [186]. ^225^Ac-lintuzumab was then tested in 40 older patients. Objective responses were observed in nine and six patients receiving doses of 2.0 μCi/kg and 1.5 μCi/kg, respectively. Although myelosuppression was observed in all patients, the study has thus far concluded that fractionated-dosing of ^225^Ac-lintuzumab can be combined safely with cytarabine and has antileukemic activity [187].

### 5.3. Ongoing Clinical Trials of RIT in AML

A number of ongoing clinical trials of RIT for AML are at various stages (Table 4). The SIERRA trial (NCT 02665065) is the first randomized Phase III study comparing a conventional myeloablative conditioning regimen plus allogeneic HCT versus ^131^I-BC8 plus HCT. The trial was specifically designed to enroll an estimated 150 patients >55 years old with R/R AML. Because the SIERRA trial is randomized, if patients in the conventional treatment arm do not respond to conventional treatment, the trial allows for crossover for patients to receive ^131^I-BC8 as well. Findings from the first 25% (*n* = 38) of patients enrolled demonstrated the feasibility of the trial. [181] At randomization, the median ranges for bone marrow blasts in the investigational and control arms were 30% (range, 4–74%) and 26% (range, 6–97%). Eighteen of 19 patients treated with ^131^I-BC8 successfully engrafted with a median of 13 days (range, 9–22). Fifteen patients (79%) receiving conventional therapy did not achieve complete remission, and of these, 10 crossed over to receive ^131^I-BC8 and were able to undergo HSCT. Thus far, hematologic and nonhematologic toxicities have been similar between arms. However, NRM was observed in patients who were randomized to the ^131^I-BC8 arm. Further updates are planned for 50% and 75% of enrolled patients.

For α-particle RIT, a multicenter phase 1/2 trial (NCT 03867682) is in the planning stages to recruit patients ≥18 years of age with R/R AML. The trial will determine the MTD and the overall response of ^225^Ac-lintuzumab when combined with the drugs venetoclax. Recently, the B-cell lymphoma 2 (BCL-2) protein was demonstrated to play a central role in the survival of AML cells [188]. Venetoclax, an inhibitor of BCL-2, has recently been demonstrated in a Phase Ib dose-escalation study in ≥65 years of age with naive AML and ineligible for conventional treatment had favorable outcomes [189]. In November 2018, the U.S. Food and Drug Administration granted an accelerated approval to venetoclax for use in newly diagnosed AML who are 75 years or older or who have comorbidities that preclude use of intensive chemotherapy.

A Phase 1 trial (NCT 03441048) is currently recruiting patients ≥18 years of age with naive/secondary and R/R-AML to evaluate the therapeutic effectiveness of ^225^Ac-lintuzumab when combined with cladribine, cytarabine, granulocyte colony stimulating factor, and mitoxantrone (CLAG-M). Because CLAG-M has been shown to have impressive anti-leukemic activity and acceptable toxicity in young and older patients with R/R AML, it is rapidly becoming a valuable addition to chemotherapeutic options available to patients [190,191]. The primary outcomes will be to determine the MTD and the toxicities from the therapeutic combination.

FHCRC is currently recruiting patients with advanced or R/R AML who can receive ^211^At-BC8 combined with fludarabine and 2–3-Gy TBI as a preparative regimen for HLA-matched (NCT 03128034) or related haplo-identical (NCT 03670966) allogeneic HCT. Thirty participants are estimated for recruitment in each trial. The objectives will be to determine the MTD of ^211^At-BC8 and AML response.

### 5.4. Future of RIT for AML

Future optimization of RIT will require better quantitative methods to estimate the dose of radiation absorbed in critical tissues which will allow for individualizing patient treatment and further reducing toxicity. This concept has matured in recent years and is widely known as “theranostics”. As previously described, AML patients treated with ^131^I-BC8 were first imaged to visualize the distribution and estimate the absorbed radiation dose in various organs in humans. These patients were administered the same reagent, which served as both a diagnostic and therapeutic purpose. Currently, dose-escalation with radiolabeled mAbs determines the MTD based on several toxicity parameters. Despite being in the era of precision medicine, which RIT conforms with, current protocols still implement a “one-dose-fits-all” approach. As the distribution of radiolabeled mAbs is variable among patients with AML, it is vital that the accuracy for individualized organ doses be improved by being able to monitor and adjust dosages [192]. The development of more precise and streamlined methods for individualized patient dosimetric determination will increase the effectiveness of RIT for R/R AML.

It is important to note that radiolabeled mAbs undergo extensive preclinical development prior to clinical testing—a drug maturation process the National Cancer Institute (NCI) supports [193]. Kunos and colleagues [193] recently summarized the launch of the NCI Cancer Therapy Evaluation Program, which has determined that radioactive drugs are an important strategic experimental therapeutic approach for AML for patients with R/R AML. The NCI is developing organizational plans for scientific review, oversight, medical monitoring, and further infrastructure elements essential for full radioactive drug development. Commercial partners, such as Actinium Pharmaceuticals, Inc., also realize the commercialization impact with radiolabeled antibodies and further benefit the development of these agents by providing funding for clinical trials. With governmental and industrial support and with the radiolabeled mAbs ^131^I-BC8, ^225^Ac-lintuzumab, and ^211^At-BC8 currently being tested in clinical trials, RIT is on the cusp of becoming a realistic alternative for patients with R/R AML. Certainly, there is a need for further development and optimization, such as broader availability of α-emitters and delivering appropriate radiation dose to circumvent radioresistance while sparing radiosensitive tissues. Thus, we eagerly await the outcome of the active and recruiting trials to provide guidance on future directions for more effective clinical implementation.

## 6. Conclusions

In this study, we outlined the four major approaches to treat AML with antibody therapeutics. The initial development of unconjugated antibody therapy was not successful for AML possibly owing to limitations of patients’ innate immune systems to be activated by ADCC.

However, the subsequent development of Mylotarg demonstrated that antibody potency could be enhanced by conjugation to a toxin and impact clinical outcomes in subsets of patients. Another similar approach would be to add a radiolabeled molecule capable of emitting alpha or beta particles to enhance antibody potency. As more infrastructure and support for RIT becomes widespread, this modality will provide patients with relapsed or refractory AML with additional options. Finally, molecular recombination techniques have allowed numerous antibody format variations with the possibly of multivalency increasing the binding affinity, specificity or activation properties over native antibodies. It is also conceivable that some or all of the four major pathways of antibody therapeutics be combined to take advantage of the higher binding affinity that dual targeting mAbs possess and co-label them with toxins or potent radioisotopes. Furthermore, a combination of novel antibody formats with adoptive cell therapy approaches such as CAR-T cells and NK cells presents an attractive means to develop a multimodal therapy with the potential for better therapeutic efficacy.

## Figures and Tables

**Table 1:** Clinical trials of bispecific antibody therapy for AML.

NCI Clinical Trial and Phase	Target	Agent(s)	Inclusion Criteria	Estimated Start and End Dates	Outcomes	Status
NCT02715011 Phase 1	CD123	JNJ-63709178 CD123 × CD3 Duobody	≥18 years of age with R/R AML	June 2016 October 2021	MTD, ORR, 1.5 year EFS and RFS	Suspended
NCT02520427 Phase 1	CD33	AMG330 CD33 × CD3 Tandem scFv (BiTE)	≥18 years of age with R/R AML	August 2015 January 2020	MTD and ORR duration at 3 years	Suspended
NCT02152956 Phase 1	CD123	MGD006 CD123 × CD3 DART flotetuzumab	≥18 years of age with R/R AML	May 2014 April 2020	MTD and OS at 2 years	Recruiting

AML: acute myeloid leukemia; R/R: felapsed/refractory; MTD: maximum tolerated dose; ORR: overall response rate (CR + CRi); OS: overall survival; RFS: relapse free survival.

**Table 2:** Clinical trials of toxin-conjugated antibodies for AML.

NCI Clinical Trial and Phase	Target	Agent(s)	Inclusion Criteria	Estimated Start and End Dates	Status
NCT03374332 Phase 2	CD33	gemtuzmab ozogamicin	≥18 years of age with R/R AML	June 2019 March 2021	Not yet recruiting
NCT03737955 Phase 2	CD33	gemtuzmab ozogamicin	≥2 years of age with AML in CR with MRD after induction chemotherapy	November 2018 August 2021	Recruiting
NCT03531918 Phase 1/2	CD33	gemtuzmab ozogamicin in combination with GCLAM	≥18 years of age with untreated “high-grade” myeloid neoplasm (≥10% Blasts in blood or BM) or AML, exluding APL	September 2018 July 2025	Recruiting
NCT02674763 Phase 1	CD33	IMGN779	≥18 years of age with R/R AML	March 2016 December 2019	Recruiting
NCT03386513 Phase 1	CD123	IMGN632	≥18 years of age with R/R CD123 + AML and other CD123 + malignancies	January 2018 February 2021	Recruiting
NCT02864290 Phase 1	FLT3	ASP1235 (AGS62P1)	≥18 years of age with R/R AML	November 2016 January 2024	Recruiting
NCT03957915 Phase 1	CD71	INA03	≥18 years of age R/R AML, ALL, or MPAL with ≥ 20% CD71 positive blasts	September 2019 November 2021	Active, not recruiting
NCT01830777 Phase 1	CD30	Brentuximab vedotin in combination with Mitoxantrone, Etoposide, and Cytarabine	≥18 years of age with CD30 + relapsed AML	May 2013 December 2019	Active, not recruiting

NCI: National Cancer Institute; AML: Acute Myeloid Leukemia; R/R: Relapsed/Refractory; MRD: Measurable Residual Disease; BM: Bone Marrow; GCLAM: Granulocyte-Colony Stimulating Factor, Cladribine, Cytarabine and Mitoxantrone.

**Table 3:** Radionuclides used in active clinical trials for AML.

Radionuclide	T ½	Emission	Emax (keV)	Range (µm)
**β-Emitting Radionuclides (LET = 0.1–1.0 keV/µm)**				
Iodine-131	8.02 days	β and γ	610/362	2300
Yttrium-90	2.67 days	β	2250	11,300
**α-Emitting Radionuclides (LET = 50–230 keV/µm)**				
Astatine-211	7.2 h	α and X	5870 and 7450/ 77–92	80
Actinium-225	9.92 days	4α, 2β and γ	6000–8000/ 198–659/218–444	90
Bismuth-213	45.59 min	α and γ	8400/440	17

**Table 4:** Clinical trials of RIT for AML.

NCI Clinical Trial and Phase	Target	Agent(s)	Inclusion Criteria	Estimated Start and End Dates	Outcomes	Status
NCT02665065 (SIERRA) Phase 3	CD45	^131^I-BC8 Fludarabine 2-Gy TBI	≥55 years of age with R/R AML patients	June 2015 June 2020	Durable CR and OS at 1 year	Recruiting
NCT03867682 Phase 1/2	CD33	^225^Ac-lintuzumab Venetoclax Spironolactone	≥18 years of age with refractory R/R AML.	May 2019 November 2022	MTD and complete and partial remission status at 6, 12, and 24 months	Not yet recruiting
NCT03670966 Phase 1/2	CD45	^211^At-BC8 Fludarabine Cyclophosphamide 2-Gy TBI Haplotype transplant	≥18 years of age with R/RAML who have an available haploindentical donor for a haplo HSCT.	March 2019 September 2024	Toxicity (GVHD, and NRM), donor chimerism, rate of engraftment, and OS up to 100 days and maintenance of remission at 2 years	Recruiting
NCT03128034 Phase 1/2	CD45	^211^At-BC8 Fludarabine 2-3-Gy TBI Haplotype transplant	≥18 years of age with R/R AML who have an available haploindentical donor for a haplo HSCT.	October 2017 March 2023	Toxicity (GVHD, and NRM), donor chimerism, rate of engraftment, and OS up to 100, remission at 2 years	Recruiting
NCT03441048 Phase 1	CD45	^211^At-BC8 CLAG-M (cladribine, cytarabine, G-CSF, mitoxantrone)	≥18 years of age with R/R AML	May 2018 October 2020	MTD and toxicity	Recruiting

AML: acute myeloid leukemia; R/R: relapsed/refractory; MTD: maximum tolerated dose; OS: overall survival; GVHD: graft versus host disease; NRM: non-relapse mortality.
